# C, H, O, N Stable Isotope Analysis Coupled with Chemometrics for Geographic Origin Authentication of Pacific White Shrimp (*Litopenaeus vannamei*) in China

**DOI:** 10.3390/foods15081274

**Published:** 2026-04-08

**Authors:** Na Wang, Caixia Wang, Huiyu Wang, Lang Zhang, Min Zhang, Hongli Jing, Lin Mei, Songyin Qiu, Xiaofei Liu, Jizhou Lv, Shaoqiang Wu

**Affiliations:** 1 Chinese Academy of Quality and Inspection & Testing, Beijing 100176, China; wangna85@yeah.net (N.W.);; 2 Technology Innovation Center of Animal and Plant Product Quality, Safety and Control, State Administration for Market Regulation, Beijing 100176, China; 3 Yangtze River Fisheries Research Institute, Chinese Academy of Fishery Sciences, Wuhan 430223, China

**Keywords:** pacific white shrimp, *Litopenaeus vannamei*, stable isotope analysis, food traceability, origin authentication, food safety

## Abstract

Pacific white shrimp (*Litopenaeus vannamei*) is a major aquaculture product worldwide. For consumers, discriminating domestic from imported sources of shrimp meat, and individual domestic sources, can be highly desirable because of the different meat quality and environmental contamination from geographically different origins of shrimp. This study evaluated the potential of stable isotope analysis (δ^13^C, δ^15^N, δ^2^H, δ^18^O) with chemometric models to authenticate the origins of Pacific white shrimp sold in China. Shrimp samples from domestic (Guangxi, Fujian, Shandong, Inner Mongolia) and foreign (Ecuador) sources were analyzed, using statistical analyses. The four-isotope model achieved 89.3% cross-validation accuracy in distinguishing domestic and foreign shrimp, with an overall prediction Area Under the Curve (AUC) of 0.901 (95% CI: 0.819–0.983)—significantly outperforming single-isotope models. Differences in δ^13^C and δ^15^N reflected feed source variations, while δ^2^H and δ^18^O (Variable Importance in the Projection (VIP) > 1, key discriminatory indicators) mirrored geographic environmental difference. Although δ^15^N did not differ significantly among groups, the combination of all four isotopes reduced limitations of individual δ^2^H/δ^18^O use. This approach enhanced the precision, reliability, and applicability of stable isotope analysis for origin authentication by leveraging complementary isotopic data and robust statistical frameworks. These findings demonstrate the proposed model’s potential as a cost-effective, copyright-compliant framework for shrimp origin authentication, with implications for isotopic traceability across food science fields.

## 1. Introduction

Pacific white shrimp (*Litopenaeus vannamei*) is a globally popular aquatic food product, valued for its low-fat, low-cholesterol, and high-quality protein content, as well as its high meat yield compared to other shrimp species [1,2,3,4,5]. China is one of the world’s major producers of Pacific white shrimp; however, domestic consumption far exceeds production, leading to a heavy reliance on imports to fill the supply gap [6]. This dependence on both domestic and imported sources has heightened consumer demand for reliable origin authentication—consumers seek to distinguish between domestic and imported shrimp, as well as among individual domestic sources, due to concerns about meat quality variations, environmental contamination risks, and compliance with food safety standards [7,8,9]. Notably, public awareness of seafood mislabeling remains low, but surveys show that most consumers express strong concern once informed of such issues and prefer seafood with independently verified origin labels [10].

Reliable origin tracing of shrimp is critical to addressing these concerns. Verifying the geographical origin and aquaculture patterns of imported Pacific white shrimp can improve labeling authenticity, enhance food safety, and strengthen consumer trust. To achieve this, comprehensive elemental profiling techniques are needed to characterize shrimp from different sources, with insights that can further inform shrimp production practices—including productivity optimization, quality control, environmental stewardship, and market transparency [6,11].

Stable isotope analysis has emerged as a robust tool for food origin tracing, as isotope ratios in heterotrophic organisms directly reflect the environmental conditions and food sources of their habitats [12,13,14]. This tool is grounded in core principles of isotope ecology and trophic dynamics, where isotopic signatures in aquatic organisms integrate dietary sources and trophic fractionation effects across food webs [15]. For hydrogen (δ^2^H) and oxygen (δ^18^O) isotopes, their spatial variability in aquatic systems is further governed by global precipitation isotope patterns, which are shaped by latitude, atmospheric circulation, and evaporation intensity—forming a natural geographic fingerprint for aquatic product traceability [16,17].

This latitudinal and hydrological control of precipitation isotopes is a well-established geochemical principle first defined by the classic Dansgaard effect [16], and global precipitation isotope models have further quantified the predictable spatial variability of δ^2^H and δ^18^O across latitudes and climatic zones [17]. Stable isotopes have since become a gold standard for food geographic origin authentication, with δ^13^C/δ^15^N tracing trophic dynamics and dietary sources and δ^2^H/δ^18^O recording habitat geochemical characteristics [12], a dual utility that makes multi-isotope integration particularly powerful for aquatic product traceability [18].

In agriculture and aquaculture, stable isotopes (e.g., δ^13^C, δ^15^N) have been widely used to verify the origin and farming patterns of aquatic animals [5,6,19,20,21]. For shrimp specifically, prior studies have highlighted the potential of isotopic markers: for example, trace element analysis revealed higher mineralization in Ecuadorian shrimp compared to Asian counterparts [22], and δ^15^N and δ^34^S have been identified as key indicators of feed sources and habitat characteristics [5]. However, two critical gaps remain in existing research: first, most studies focus on single or dual isotopes, ignoring the complementary information from hydrogen (δ^2^H) and oxygen (δ^18^O) isotopes—markers that directly reflect water source variations (e.g., latitude-driven precipitation isotope effects, coastal vs. inland evaporation differences) [6,23]; second, existing analyses often rely on commercial statistical software (e.g., SPSS), which poses copyright risks and limits method reproducibility in cross-institutional or international collaborations.

Recent advances in food traceability have demonstrated the value of combining stable isotope analysis with chemometric models [5,24,25,26]. Building on this, the present study establishes a novel, copyright-compliant framework for Pacific white shrimp origin authentication by integrating four stable isotopes (δ^13^C, δ^15^N, δ^2^H, δ^18^O) with open-source statistical tools. The four-isotope model leverages the synergistic effects of carbon and nitrogen (reflecting feed sources) and hydrogen and oxygen (reflecting geographic water conditions), addressing the limitations of single-isotope approaches. Statistical analyses (including ANOVA, linear discriminant analysis [LDA], and binary logistic regression) were performed using PSPP 2.0.0 (GNU Project), an open-source software fully compatible with conventional commercial tools, ensuring both result reliability and compliance with academic copyright standards.

This study focused on shrimp from five key sources: four domestic provinces in China (Guangxi, Fujian, Shandong, and Inner Mongolia) and one major import source (Ecuador). Notably, the domestic samples span diverse aquaculture environments—coastal marine (Shandong, Fujian, Guangxi) and inland freshwater (Inner Mongolia)—while Ecuador represents a typical tropical marine import source [27,28]. By quantifying isotopic differences and their links to geographic factors (e.g., latitude, water type, evaporation intensity), this study aims to provide a proof-of-concept for a cost-effective, reproducible method for authenticating shrimp origins. The findings will strengthen China’s aquaculture traceability protocols and food safety systems—areas of growing interest in both academic and industrial contexts [5,29,30,31]—and offer a scalable framework for isotopic traceability across other food products.

## 2. Materials and Methods

### 2.1. Materials

All reagents were analytically pure. Chloroform was purchased from Kemio Chemical Reagent Ltd. (Tianjin, China). Methanol was purchased from Fisher Scientific Ltd. (Waltham, MA, USA).

### 2.2. Sample Collection

**Figure 1:**
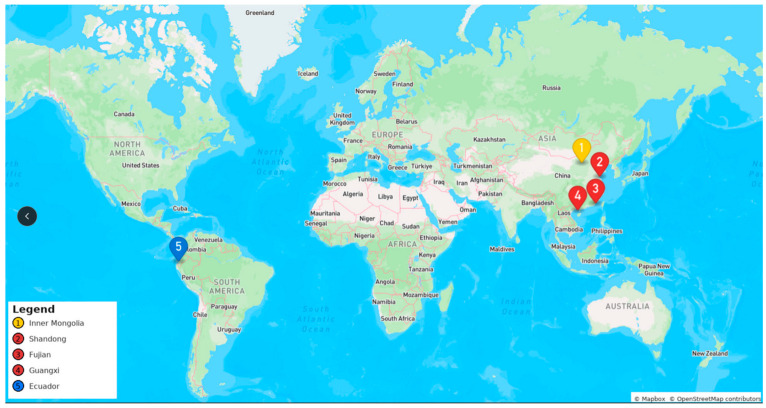
Map of sample locations. The yellow marker (1) indicates the sampling site in Inner Mongolia, China; red markers (2, 3, 4) indicate sampling sites in Shandong, Fujian, and Guangxi, China, respectively; the blue marker (5) indicates the sampling site in Ecuador. National border lines are presented to clarify the geographic distribution of sampling locations.

Pacific white shrimp (*L*. *vannamei*) samples were collected from five origins between June and August 2024: four domestic provinces in China from south to north (Shandong, Fujian, Guangxi, Inner Mongolia) and one major import source (Ecuador) (Table 1). For each origin, 30 samples (commercial size: 15–20 g/individual) were randomly selected from local aquaculture farms (domestic) or import ports (Ecuador), with three biological replicates per sample (biological replicates refer to three independent healthy individual shrimp collected from the same origin) (Figure 1). All replicate samples were analyzed independently, and the average values of the three replicates were used for subsequent statistical analysis.

Shandong, Fujian, and Guangxi are major producing areas for *L. vannamei* within China, and Inner Mongolia is a province where inland aquaculture has expanded significantly in recent years [30]. Ecuador was selected as the country for imported shrimp samples because it is one of the largest sources of *L. vannamei* imports into China [28]. Furthermore, Inner Mongolia is an inland freshwater pond-based aquaculture site, whereas the other four locations are marine aquaculture sites. The specimens collected from the four locations within China (Figure 1) were categorized as the Domestic group (120 samples total), and the samples from Ecuador were categorized as the Foreign group (30 samples total) for comparative analysis.

All samples were collected during a similar time frame. Any seasonal or temporal differences in collection locations are potentially attributable to variations in latitude and climate conditions. Key geographic environmental data for sampling sites were obtained from: Latitude/longitude, National Geospatial Data Cloud (https://www.gscloud.cn/, China) and GeoNames (https://www.geonames.org/, Ecuador). Annual evaporation, WorldClim v2.1 database (https://worldclim.org/, spatial resolution: 30 arc-seconds) and China Meteorological Administration (CMA) annual reports (2024). Water type (freshwater/marine), Confirmed via on-site surveys (domestic) and Ecuadorian aquaculture association records (foreign), with Inner Mongolia identified as inland freshwater and others as coastal/marine (Table 2).

All collected shrimp specimens had a body length greater than 10 cm and an average body weight greater than 10 g. All shrimp samples used in this study were naturally deceased after harvest and frozen at −20 °C until further pretreatment. All sample collection and handling procedures were reviewed and approved by the Animal Care and Use Committee of Chinese Academy of Quality Inspection and Testing (Approval No.: CAQIT-AQ-202202), fully complying with national and institutional ethical guidelines for animal research.

### 2.3. Sample Pretreatment

To dry shrimp, the tail of the shrimp was cut off with scissors and the shrimp shell and line were removed. Then, 2.0 g of shrimp meat per sample was weighed with an electronic balance (BS224; Sartorious, Göttingen, Germany) and then evenly spread in a glass dish (60 mm in diameter). Finally, the glass dish with the sample was placed in a drying oven at 55 °C (FD260; PENTAX, Hamburg, Germany) for 48 h until constant weight (weight change < 0.001 g) was achieved.

Dried shrimp meat samples were ground for 5 min using a grinding mill to obtain dried shrimp powder. First, 0.15 g of powder was placed in a 2 mL centrifuge tube, and chloroform: methanol solution (2:1) was added at a solid-to-liquid ratio of 1:5. The samples were then vortexed for 10 min, followed by centrifugation at 5000 rpm for 5 min. The supernatant was removed using a micropipette. After repeating the extraction step twice, the moist defatted samples were dried in a drying oven at 60 °C for 3 h until constant weight was achieved. Finally, the samples were ground for 1 min using the grinding mill to prepare defatted shrimp powder for subsequent analysis.

### 2.4. Isotope Mass Spectrometry Analysis

The following analyses were carried out on the basis of a previously described method [32].

δ^13^C and δ^15^N sample determination: 300 µg of dried defatted shrimp powder was weighed, wrapped in a tin capsule, and introduced into an Elemental Analyzer coupled with an Isotope Ratio Mass Spectrometer (EA-IRMS, Flash2000, Thermo Finnigan, Bremen, Germany) for carbon and nitrogen stable isotope ratio analysis. Samples entered the elemental analyzer via an autosampler, where carbon and nitrogen were oxidized at 960 °C to form CO_2_ and NO_x_ gases. These gases were then passed over hot copper wire to reduce NO_x_ to N_2_. Carrier gas (high-purity helium) and oxygen entered the gas chromatographic column at 50 °C to separate CO_2_ and N_2_. The gases were transferred to a ConFlo IV universal continuous flow interface (Thermo Finnigan, Bremen, Germany). The carrier gas (high-purity helium) flow rate was 100 mL/min, and the oxygen injection speed was 175 mL/min. Subsequently, C and N stable isotope ratios were analyzed using an isotope ratio mass spectrometer (DELTA V Advantage, Thermo Finnigan, Bremen, Germany). Data were calibrated against the following international reference materials: USGS62, USGS42, and IAEA-600. δ^13^C values were reported relative to V-PDB and δ^15^N values were reported relative to atmospheric N_2_. For δ^13^C and δ^15^N values, two-point linear normalization was performed using IAEA-600 and USGS62, with secondary correction using USGS42. The precisions of C and N were *<*0.15 ‰.

δ^2^H and δ^18^O sample determination: 150 µg of dried defatted shrimp powder was weighed, wrapped in a silver capsule, and introduced into the EA-IRMS for hydrogen and oxygen stable isotope ratio analysis. Samples entered the elemental analyzer via an autosampler, where hydrogen and oxygen were pyrolyzed at 1380 °C and converted to H_2_ and CO by glassy carbon in the pyrolysis furnace. H_2_ and CO were separated by gas chromatography with the column temperature at 80 °C. H_2_ and CO were then introduced into the isotope ratio mass spectrometer via ConFlo IV to measure H and O stable isotope ratios. The carrier gas (high-purity helium) flow rate was 100 mL/min, and oxygen injection speed was 175 mL/min. Data were calibrated against the following international reference materials: USGS90, CBS, KHS. For δ^2^H and δ^18^O values, two-point linear normalization was performed using CBS and USGS90, with secondary correction using KHS. The precision of H was *<*3 ‰, and O’s was *<*0.4 ‰.

Analytical precision was calculated using the relative standard deviation of the measurement results of the five standard samples. Stable isotope analysis results were expressed in δ (‰) and calculated as follows:

δ (‰) = [(R_sample − R_standard)/R_standard] × 1000

where R_sample and R_standard are the stable isotope ratios of the sample and the international standard material, respectively. Carbon, nitrogen, hydrogen, and oxygen stable isotope ratios are denoted as δ^13^C (‰), δ^15^N (‰), δ^2^H (‰), and δ^18^O (‰), respectively.

### 2.5. Data Analysis

#### 2.5.1. Uncertainty Assessment

To ensure the reliability and traceability of isotopic data and model parameters, a com prehensive uncertainty assessment was conducted for all stable isotope measurements and statistical model parameters, following the guidelines of the International Organization for Standardization (ISO/IEC) 17025 [33] and IAEA technical reports for stable isotope analysis.

#### 2.5.2. Statistical Analysis Implementation

PSPP 2.0.0 (GNU Project, https://www.gnu.org/software/pspp/ (accessed on 28 May 2025)) was used for one-way analysis of variance (ANOVA) with Duncan’s multiple range test, LDA, and binary logistic regression. PSPP is an open-source statistical software that maintains full compatibility with IBM SPSS (version 22.0) in terms of data format (.sav file) and syntax logic; thus, all statistical operations in this study (including ANOVA for isotopic difference comparison, LDA for discriminant formula construction, and logistic regression for domestic/foreign sample classification) were implemented following the same workflow as SPSS, ensuring consistent results with conventional commercial statistical tools.

Prior to the application of ANOVA and LDA, mandatory statistical assumption tests were conducted to validate the model applicability: the Shapiro–Wilk test confirmed that all isotopic data conformed to a normal distribution, and Levene’s test verified the homogeneity of variances across all groups. For all predictive and discriminant models (ANOVA-LDA, binary logistic regression, and OPLS-DA), 10-fold cross-validation was adopted to evaluate model performance and avoid overfitting, a robust and widely accepted cross-validation strategy in chemometric analysis for food traceability research. Additionally, the Variance Inflation Factor (VIF) test was performed to assess multicollinearity among the four isotope variables (δ^13^C, δ^15^N, δ^2^H, δ^18^O) in LDA and binary logistic regression models; all calculated VIF values were <3, indicating the absence of significant multicollinearity and ensuring the reliability of the model coefficients and discriminant results.

All data were preprocessed with Pareto scaling in SIMCA 14.1 (Umetrics AB, Umeå, Sweden) for Principal Component Analysis (PCA), Orthogonal Partial Least Squares Discriminant Analysis (OPLS-DA), and Hierarchical Cluster Analysis (HCA) analysis. OPLS-DA was used to obtain the classifying model and extract variables that had important contributions to the classification, and HCA was used to infer natural groupings within the dataset, and plots were constructed. HCA was performed using Ward’s linkage method and Euclidean distance [34].

Key discriminative features were identified from all variables using multivariate statistical techniques, such as PCA and LDA. Measured variables were compiled into a data matrix, which was standardized through column-wise normalization and mean centering to ensure consistency. Signal noise was then reduced, dimensionality was decreased, and unsupervised PCA was applied. Finally, a supervised LDA model was developed using a subset of orthogonal principal components instead of the original variables, and its performance was assessed through cross-validation. Significance for all analyses was set to *p* < 0.05.

## 3. Results

### 3.1. ANOVA

ANOVA with Duncan’s multiple range test revealed significant differences (*p* < 0.05) in the carbon (δ^13^C), nitrogen (δ^15^N), hydrogen (δ^2^H), and oxygen (δ^18^O) stable isotope ratios among shrimp meat samples from the five origins (Shandong, Fujian, Guangxi, Ecuador, and Inner Mongolia) (Table 1). Specifically, δ^13^C values ranged from −23.16 ± 0.17‰ (Ecuador) to −22.08 ± 0.25‰ (Inner Mongolia), with Inner Mongolia samples significantly enriched in ^13^C compared to all other groups; δ^15^N values were highest in Inner Mongolia (5.90 ± 0.38‰) and lowest in Shandong (4.42 ± 0.79‰); low-latitude climatic characteristics lead to elevated δ^2^H values in precipitation. Correspondingly, δ^2^H values showed a distinct latitudinal gradient, decreasing from −86.70 ± 3.84‰ in Shandong (35°24′59″ N) to −104.27 ± 2.13‰ in Inner Mongolia (40°39′01″ N); δ^18^O was most enriched in coastal Guangxi (23.93 ± 5.39‰) and Fujian (22.74 ± 2.32‰), and most depleted in inland Inner Mongolia (17.57 ± 0.67‰) (Table 2).

**Table 1:** Significant differences of four stable isotopes in shrimp meat from five locations based on ANOVA with Duncan’s multiple range test.

Stable Isotope	Shandong	Fujian	Guangxi	Inner Mongolia	Ecuador
δ^13^C (‰)	−23.05 ± 0.33 ^e^	−22.98 ± 0.25 ^c^	−22.67 ± 0.23 ^b^	−22.08 ± 0.25 ^a^	−23.16 ± 0.17 ^d^
δ^15^N (‰)	4.42 ± 0.79 ^c^	5.19 ± 0.31 ^b^	5.20 ± 0.37 ^b^	5.90 ± 0.38 ^a^	5.43 ± 0.58 ^b^
δ^2^H (‰)	−86.70 ± 3.84 ^a^	−97.92 ± 2.09 ^c^	−101.19 ± 2.25 ^d^	−104.27 ± 2.13 ^e^	−94.50 ± 4.99 ^b^
δ^18^O (‰)	19.47 ± 0.90 ^b^	22.74 ± 2.32 ^a^	23.93 ± 5.39 ^a^	17.57 ± 0.67 ^c^	19.05 ± 0.71 ^b^

Note: 1. Data are shown as mean ± standard deviation (SD, *n* = 30) with relative standard deviation (RSD) in percentage; RSD = (SD/mean) × 100%, reflecting the measurement variability of isotopic values. Relative standard deviation (RSD) for each isotope (reflecting data variability): δ^13^C (0.73–1.43%), δ^15^N (5.97–17.87%), δ^2^H (2.04–5.28%), δ^18^O (3.73–22.52%). 2. Expanded uncertainty (U, k = 2, 95% CI) for all isotopic data was calculated, with instrumental precision (SD) of δ^13^C < 0.15‰, δ^15^N < 0.15‰, δ^2^H < 3.0‰, δ^18^O < 0.4‰. 3. Different lowercase superscript letters indicate significant differences between groups (*p* < 0.05) based on ANOVA with Duncan’s multiple range test, implemented in PSPP 2.0.0.

**Table 2:** Correlation between stable isotopes and geographic environmental factors of shrimp sampling sites.

Sampling Site	Water Type	Latitude	Annual Evaporation (mm)	δ^13^C (‰)	δ^15^N (‰)	δ^2^H (‰)	δ^18^O (‰)
Shandong	Coastal marine	35°24′59″ N	~1200	−23.05 ± 0.33 ^e^	4.42 ± 0.79 ^c^	−86.70 ± 3.84 ^a^	19.47 ± 0.90 ^b^
Fujian	Coastal marine	24°30′47″ N	~1600	−22.98 ± 0.25 ^c^	5.19 ± 0.31 ^b^	−97.92 ± 2.09 ^c^	22.74 ± 2.32 ^a^
Guangxi	Coastal marine	21°28′28″ N	~1800	−22.67 ± 0.23 ^b^	5.20 ± 0.37 ^b^	−101.19 ± 2.25 ^d^	23.93 ± 5.39 ^a^
Inner Mongolia	Inland freshwater	40°39′01″ N	~800	−22.08 ± 0.25 ^a^	5.90 ± 0.38 ^a^	−104.27 ± 2.13 ^e^	17.57 ± 0.67 ^c^
Ecuador	Tropical marine	02°11′39″ S	~2000	−23.16 ± 0.17 ^d^	5.43 ± 0.58 ^b^	−94.50 ± 4.99 ^b^	19.05 ± 0.71 ^b^

Note: 1. Different lowercase superscript letters indicate significant differences between groups (*p* < 0.05), with values sharing the same letter indicating no significant difference, and different letters indicating a statistically significant difference.

These isotopic differences were not random but closely linked to the geographic environmental characteristics of each sampling site. Table 2 systematically integrates the stable isotope data (from Table 1) with key geographic factors (latitude, water type, annual evaporation), further verifying that geographical origin drives the isotopic signatures of shrimp meat. For instance:

The highest δ^13^C and δ^15^N values of Inner Mongolia were attributed to its freshwater aquaculture system—feed primarily relies on C3 plants (e.g., terrestrial grains) with higher δ^13^C, while agricultural activities (e.g., fertilizer use) in the watershed increase nitrogen input, leading to elevated δ^15^N in shrimp; the lowest δ^2^H and δ^18^O result from high latitude (reducing precipitation δ^2^H) and low evaporation (minimizing ^18^O enrichment in water).

δ^18^O values of Shandong increased with decreasing latitude and increasing evaporation (Guangxi: 21°28′28″ N, ~1800 mm evaporation; Shandong: 35°24′59″ N, ~1200 mm evaporation), as intense coastal evaporation preferentially removes light ^16^O from water, enriching residual water (and thus shrimp tissues) in ^18^O.

Low δ^13^C (−23.16 ± 0.17‰) of Ecuador reflects the dominance of marine carbon sources in tropical oceans, while moderate δ^2^H (−94.50 ± 4.99‰) balances the effects of low latitude (elevating precipitation δ^2^H) and high marine evaporation (slightly depleting δ^2^H).

Additionally, significant differences (*p* < 0.05) in all four isotopes were observed between the Domestic group (Shandong, Fujian, Guangxi, Inner Mongolia) and the Foreign group (Ecuador), further confirming that geographical origin is a key determinant of shrimp stable isotope characteristics. These findings, combined with the mechanistic links in Table 2, lay a critical foundation for subsequent origin authentication models—stable isotopes act as “biological fingerprints” of geographic environments, enabling reliable differentiation of shrimp sources.

### 3.2. LDA

The application potential of stable isotopes for origin traceability was further validated by LDA models constructed using the four isotope indicators (δ^13^C, δ^15^N, δ^2^H, δ^18^O), which not only demonstrated high distinguishability between shrimp meat from different origins but also tightly linked the discriminant ability to geographic environmental differences (Table 2 and Table 3).

#### 3.2.1. Construction of LDA Discriminant Functions

Linear discriminant formulas for the five shrimp groups (Shandong, Fujian, Guangxi, Ecuador, Inner Mongolia) were established based on the four stable isotopes, with each formula quantitatively integrating the contribution of isotopic signatures shaped by geographic factors (Table 3). The discriminant formula for Inner Mongolia (−493.495 × δ^13^C + 135.338 × δ^15^N − 5.763 × δ^2^H + 9.553 × δ^18^O − 6232.473) assigns a larger absolute coefficient to δ^2^H (−5.763) and δ^13^C (−493.495), reflecting the critical role of these two isotopes in distinguishing its freshwater habitat (high δ^13^C from C3 feeds, low δ^2^H from high latitude) from other marine sites (Table 2). The formula for Ecuador (−516.991 × δ^13^C + 140.245 × δ^15^N − 4.698 × δ^2^H + 10.031 × δ^18^O − 6695.008) emphasizes δ^13^C (coefficient: −516.991), consistent with its unique tropical marine carbon source (low δ^13^C, Table 2) that differentiates it from domestic marine shrimp.

The measurement uncertainty of isotopic data and the statistical uncertainty (standard error, SE; 95% confidence interval, CI) of all model parameters (LDA discriminant coefficients, binary logistic regression AUC, classification thresholds) were quantified to evaluate their impact on the reliability of origin discrimination models (Table 1 and Table 4). For the LDA discriminant coefficients (Table 3), statistical quantification revealed that the key discriminatory isotopes (δ^13^C and δ^2^H) exhibited low standard error (SE < 10.0) and narrow 95% CI, with no zero values within the CI range—indicating that the coefficient estimates for these two isotopes were statistically significant and less affected by isotopic measurement variability. In contrast, the coefficients of δ^15^N and δ^18^O showed slightly higher SE but still no zero within the 95% CI, confirming their non-negligible contribution to group discrimination.

The low uncertainty of δ^13^C and δ^2^H coefficients is consistent with the low measurement variability (low RSD, Table 1) of these two isotopes across samples, and this consistency ensures that the LDA discriminant model can stably capture the geographic differences in shrimp isotopic signatures. Additionally, the multicollinearity test (VIF < 3, Section 2.5.2) eliminated the systematic error caused by variable correlation, further reducing the uncertainty of coefficient estimation and enhancing the robustness of the LDA discriminant formulas for practical origin authentication. For the binary logistic regression model, the SE and 95% CI for AUC and optimal classification thresholds (core evaluation parameters) are presented in Table 4, which directly reflects the statistical reliability of the domestic/foreign shrimp discrimination results.

#### 3.2.2. Classification Accuracy of LDA Models

Based on the LDA discriminant functions, the original classification accuracy for the five groups was 83.3%, with a cross-validation accuracy of 81.3% (Table 5). The high accuracy of Inner Mongolia samples (100% correct classification in both original and cross-validation, Table 5) further validated the model’s sensitivity to geographic water type: its distinct freshwater isotopic signature (high δ^13^C/δ^15^N, low δ^2^H/δ^18^O, Table 2) minimized overlap with marine groups, resulting in perfect discrimination.

For the binary classification of “Domestic vs. Foreign” shrimp, the analysis was performed on the data from domestic samples (n = 120) and foreign samples (n = 30) using binary logistic regression in PSPP. The regression equation of the established model was as follows:

X = −4.882 × A + 2.835 × B + 0.012 × C − 0.53 × D − 117.198

Prob (Foreign Sample) = 1/(1 + e^−x^)

where A, B, C, and D represent the values of δ^13^C‰, δ^15^N‰, δ^2^H‰, and δ^18^O‰, respectively. Prob (Foreign Sample) refers to the probability that a sample is a foreign one. The optimal cutoff value of the binary logistic regression model was determined to be 0.36483. This value was selected based on the receiver operating characteristic (ROC) curve analysis combined with the principle of maximizing the Youden’s index, corresponding to a specificity of 0.942 and a sensitivity of 0.833 for the model, with the area under the curve (AUC) of the model reaching 0.901. If Prob (Foreign Sample) is greater than 0.36483, the sample is identified as a foreign one; if it is less than 0.36483, the sample is identified as a domestic one (Table 4). Re-substitution analysis of 150 samples (120 domestic, 30 foreign) showed that 134 samples were correctly classified, with an overall cross-validation accuracy of 89.3% (Table 6). VAR00005 is a categorical assignment variable for the geographical origin of shrimp samples, where VAR00005 = 0 represents domestic shrimp samples (Shandong, Fujian, Guangxi, Inner Mongolia) and VAR00005 = 1 represents foreign shrimp samples (Ecuador). This assignment rule was applied to the establishment of the binary logistic regression model and the validation analysis of subsequent prediction results (Table 6). Specifically, the domestic samples (VAR00005 = 0) had a 96.7% correct prediction rate (116/120), while the foreign samples (VAR00005 = 1) had a 60% correct prediction rate (18/30), with partial misclassification attributed to overlapping marine environments.

#### 3.2.3. Practical Value of LDA Discriminant Formulas

The LDA discriminant formulas (Table 3) provide a direct operational tool for origin authentication. For unknown shrimp samples, substituting δ^13^C, δ^15^N, δ^2^H, and δ^18^O values into the five group-specific formulas yields discriminant scores; the group with the highest score is identified as the sample’s origin.

### 3.3. Multivariate Statistical Analysis

**Figure 2:**
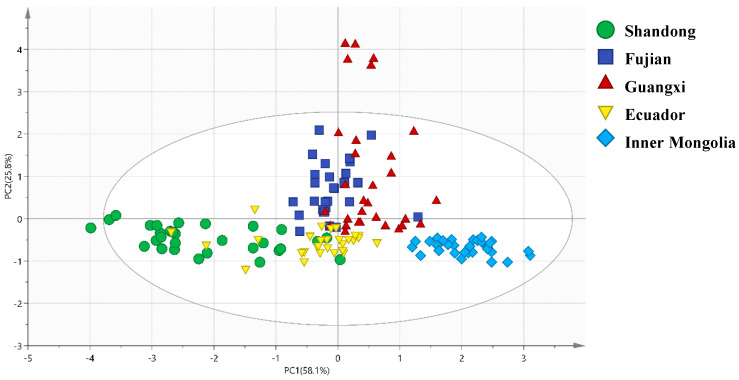
PCA score plot of stable isotope data for the five shrimp groups. PC1 (58.1% of total variance) is mainly loaded with δ^13^C and δ^15^N; PC2 (25.8% of total variance) is mainly loaded with δ^2^H and δ^18^O. Each point represents one sample, and the 95% confidence ellipses are added for each group.

**Figure 3:**
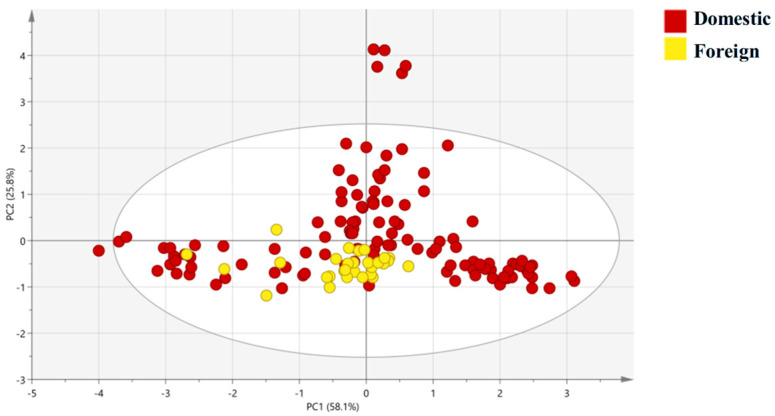
PCA plot of stable isotope data for Domestic and Foreign shrimp groups. PC1 (58.1% of total variance) is mainly loaded with δ^13^C and δ^15^N; PC2 (25.8% of total variance) is mainly loaded with δ^2^H and δ^18^O. Each point represents one sample, and the 95% confidence ellipses are added for each group.

**Figure 4:**
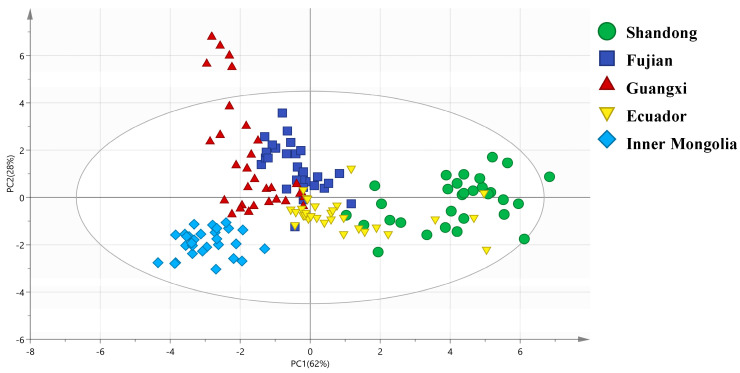
OPLS-DA score plot of stable isotope data for the five shrimp groups. PC1 (62% of total variance) is mainly loaded with δ^13^C and δ^15^N; PC2 (28% of total variance) is mainly loaded with δ^2^H and δ^18^O. Each point represents one sample, and the 95% confidence ellipses are added for each group.

**Figure 5:**
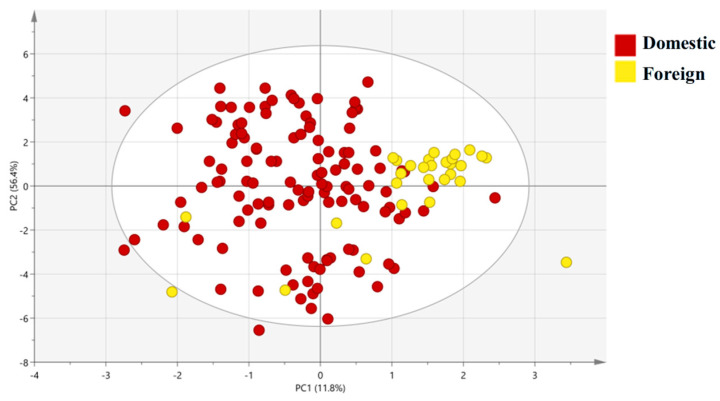
OPLS-DA score plot of stable isotope data for Domestic and Foreign shrimp groups. PC1 (11.8% of total variance) is mainly loaded with δ^13^C and δ^15^N; PC2 (56.4% of total variance) is mainly loaded with δ^2^H and δ^18^O. Each point represents one sample, and the 95% confidence ellipses are added for each group.

To further visualize the origin-related isotopic patterns and validate the synergistic effects of the four isotopes (δ^13^C, δ^15^N, δ^2^H, δ^18^O), PCA, OPLS-DA, and HCA were performed. The results not only confirmed the distinct grouping of shrimp from different origins but also revealed clear links between clustering patterns and geographic environmental factors, as summarized in Figure 2, Figure 3, Figure 4 and Figure 5 and supported by Table 2.

#### 3.3.1. Principal Component Analysis (PCA)

The PCA score plot (Figure 2) explained 83.9% of the total variance in the isotopic data via the first two principal components (PC1: 58.1%, PC2: 25.8%), providing a holistic view of sample distribution. Key observations aligned with geographic environmental differences (Table 2): Inner Mongolia samples (inland freshwater, 40° N, low evaporation) formed a tight, isolated cluster in the lower-right quadrant of the PCA plot. This separation was primarily driven by PC1 (loaded heavily with δ^13^C and δ^15^N) and PC2 (loaded with δ^2^H and δ^18^O)—Inner Mongolia’s high δ^13^C/δ^15^N (from C3 feed and agricultural N input) and low δ^2^H/δ^18^O (from high latitude and low evaporation) distinguished it from all other groups, reflecting the unique isotopic signature of its freshwater habitat. Coastal domestic samples (Fujian, Guangxi; marine environment, variable latitude/evaporation) clustered in the upper-right to upper-middle quadrants, with partial overlap between Fujian and Guangxi. This overlap was attributed to their similar geographic conditions: both are subtropical coastal regions with high evaporation (Fujian: ~1600 mm, Guangxi: ~1800 mm) and marine carbon sources, leading to comparable δ^18^O (22.74‰ vs. 23.93‰) and δ^13^C (−22.98‰ vs. −22.67‰) values (Table 2). Ecuador samples (tropical marine, 02° S, high evaporation) formed a discrete cluster between domestic coastal and Inner Mongolia groups. Their positioning was driven by low δ^13^C (−23.16‰, tropical marine carbon) and moderate δ^2^H (−94.50‰, balancing low latitude and high evaporation), as shown in Table 2, which separated them from domestic marine samples (e.g., Guangxi’s δ^13^C: −22.67‰, δ^2^H: −101.19‰).

The PCA plot for domestic vs. foreign groups shows that PC1 (58.1% variance explained) and PC2 (25.8% variance explained) show partial separation between the Domestic group (Shandong, Fujian, Guangxi, Inner Mongolia) and Foreign group (Ecuador), with minor overlap between Ecuador and domestic coastal samples (Figure 3). The result reveals a discernible separation trend along PC1: the domestic group exhibits a wider isotopic distribution (encompassing both freshwater and coastal environments), while Ecuador (foreign group) clusters near PC1 = 0. The PCA results further validated that the four isotopes effectively capture geographic-related variability, laying the groundwork for more targeted discriminant analysis.

#### 3.3.2. Orthogonal Partial Least Squares Discriminant Analysis (OPLS-DA)

To enhance the separation of overlapping groups (e.g., Fujian vs. Guangxi) and identify key isotopic markers driving origin differences, OPLS-DA was conducted (Figure 4 and Figure 5). The OPLS-DA model exhibited excellent explanatory and predictive power for the isotopic data, with a cumulative explanatory coefficient R^2^Y = 0.91 and a predictive coefficient Q^2^ = 0.85 (Figure S1). The PCA score plot (Figure 4) explained 90% of the total variance in the isotopic data via the first two principal components (PC1: 62%, PC2: 28%), providing a holistic view of sample distribution. The OPLS-DA score plot achieved clearer separation than PCA. Inner Mongolia samples were completely isolated along the predictive component, confirming their unique freshwater isotopic signature. Ecuador samples were separated from domestic coastal groups along the orthogonal component. Partial overlap between Fujian and Guangxi was reduced.

To further validate the model robustness and rule out overfitting, a permutation test with 200 permutations was performed for shrimp samples from the five origins, and the results are presented in Figure S1. The Q^2^ values derived from the permutation test for Shandong, Fujian, Guangxi, Ecuador, and Inner Mongolia were −0.0753, −0.0374, −0.0533, −0.0541, and −0.0777, respectively, all of which were below 0.05—an indicator of no overfitting for the original OPLS-DA model. Additionally, a cross-validated analysis of variance (CVANOVA) was conducted, with a resulting *p*-value < 0.001, further verifying the statistical significance of the group separation achieved by the OPLS-DA model.

#### 3.3.3. Hierarchical Cluster Analysis (HCA)

**Figure 6:**
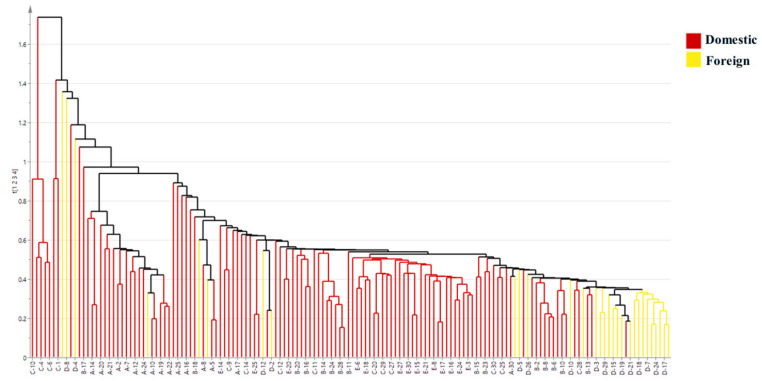
HCA plot of stable isotope data for Domestic and Foreign shrimp groups.

The HCA dendrogram for domestic vs. foreign groups shows that samples cluster into two main branches with 88% purity; only a small number of domestic coastal samples are misclustered with Ecuador (Figure 6). It demonstrates high clustering purity between the two groups. Minor misclustering corresponds to the overlap in PCA/OPLS-DA, driven by shared marine habitat traits between Ecuador and domestic coastal sites—reinforcing the need for the four-isotope model rather than single markers to achieve reliable domestic/foreign origin authentication.

#### 3.3.4. Key Isotopic Markers (VIP Values)

The variable importance in projection (VIP) scores identified δ^2^H (VIP = 1.45) and δ^18^O (VIP = 1.10) as the top discriminant indicators, followed by δ^13^C (VIP = 0.55) and δ^15^N (VIP = 0.63). The VIP values of the Domestic and Foreign groups for δ^13^C, δ^15^N, δ^2^H, and δ^18^O were 0.55, 0.44, 1.48, and 1.15, respectively. In both instances, δ^2^H and δ^18^O were key discriminatory indicators (VIP > 1), vastly outperforming δ^13^C and δ^15^N. This aligns with the geographic mechanisms in Table 2: δ^2^H and δ^18^O are direct proxies for water source and environmental conditions (latitude, evaporation), which vary most significantly across origins (e.g., Inner Mongolia’s 40° N vs. Ecuador’s 02° S, coastal evaporation vs. inland low evaporation). This indicates that hydrogen and oxygen stable isotopes are the primary differentiating elements for shrimp meat from these five origins and the main contributing factors to the differences between the Domestic and Foreign groups. For *L*. *vannamei*, the high discriminatory power of δ^2^H/δ^18^O is further enhanced by their low sensitivity to short-term dietary fluctuations compared to δ^13^C and δ^15^N [35,36], making them the core indicators for cross-regional origin differentiation as observed in this study.

These results demonstrated that the four-isotope model was able to leverage complementary isotopic data and advanced statistical methods to yield high classification accuracy for distinguishing the origin of Pacific shrimp meat.

## 4. Discussion

This study successfully established a robust and copyright-compliant framework for the geographic origin authentication of Pacific white shrimp (*L. vannamei*) by integrating four stable isotopes (δ^13^C, δ^15^N, δ^2^H, δ^18^O) with a suite of chemometric models. The four-isotope model achieved high cross-validation accuracy (81.3% for five groups, 89.3% for domestic vs. foreign), demonstrating that the synergistic use of complementary isotopic markers effectively overcomes the limitations of single-isotope approaches. Below, we interpret the key findings, contextualize them within the broader field of food traceability, address the study’s limitations, and outline future research directions.

### 4.1. Isotopic Signatures as Biological Fingerprints of Geographic Origin

The significant isotopic differences among the five groups are best explained by the distinct environmental conditions and aquaculture practices of each origin, supporting the use of these ratios as reliable “biological fingerprints” [12,23]. The high δ^13^C and δ^15^N values in Inner Mongolia samples suggest a potential link to their freshwater aquaculture system, where feeds are primarily derived from terrestrial C3 plants (e.g., corn, wheat) and agricultural nitrogen inputs are common [5,37]. In contrast, the depleted δ^13^C values in Ecuador samples are consistent with the dominance of marine phytoplankton (a C3-like carbon source with inherently low δ^13^C) in tropical coastal ecosystems [18]. These patterns align with previous studies indicating that δ^13^C and δ^15^N are robust tracers of trophic level and dietary carbon sources in aquatic organisms [19,38]; the use of δ^13^C as a dietary carbon source indicator and δ^15^N as a trophic position tracer is a foundational principle of aquatic food web ecology [15], and for *L. vannamei* specifically, tissue δ^13^C and δ^15^N have been empirically shown to faithfully reflect the nutritional contribution of different dietary sources (e.g., terrestrial C3 plants vs. marine phytoplankton) [2,39], and meta-analyses have further confirmed that crustacean isotopic fractionation factors are species-specific, reinforcing the reliability of δ^13^C/δ^15^N for tracing aquaculture feed and habitat nutrient dynamics in shrimp [40].

The systematic variations in δ^2^H and δ^18^O are indicative of the effects of latitude and evaporation processes. The association between δ^2^H and latitude aligns with the global precipitation isotope effect, whereby δ^2^H values in precipitation decline with increasing latitude [37,41]. This coupling between aquatic organism δ^2^H/δ^18^O and local precipitation isotopes is a universal pattern across freshwater and coastal marine systems [42]; for South China coastal regions (e.g., Fujian, Guangxi in this study), precipitation δ^2^H/δ^18^O variability is further amplified by monsoon and evaporation effects, with these signatures directly recorded in aquaculture pond water and subsequent shrimp tissue isotopes [43]. For *L*. *vannamei*, this environmental isotopic fidelity makes δ^2^H/δ^18^O particularly effective for distinguishing freshwater and marine aquaculture habitats as well as latitudinally distinct coastal regions [2,18], and this strong coupling between shrimp tissue δ^2^H/δ^18^O and ambient aquatic environmental factors (latitude, evaporation) has been experimentally confirmed for farmed populations [36]. Similarly, the positive relationship between δ^18^O and evaporation processes supports the established hypothesis that intense coastal evaporation enriches ambient aquatic environments—and consequently shrimp tissues—with the heavy oxygen isotope ^18^O [19]. Notably, δ^2^H and δ^18^O provide orthogonal isotopic information relative to δ^13^C and δ^15^N, facilitating the differentiation of marine shrimp samples from distinct latitudinal zones (e.g., Ecuador vs. Shandong). Combined with the dietary-driven variability of δ^13^C and δ^15^N in *L*. *vannamei* [35], these patterns reinforce that isotopic signatures integrate both habitat geochemistry and aquaculture practices, forming a comprehensive biological fingerprint for origin authentication.

It is important to emphasize that while PCA and LDA revealed strong associations between isotopic signatures and geographic factors, these statistical methods are exploratory and discriminant in nature, respectively, and do not prove causal relationships. The observed correlations provide experimental evidence for the environmental mechanisms reported in the isotope ecology literature [6,37].

### 4.2. Synergistic Effects of Multi-Isotope Integration: Overcoming Single-Isotope Limitations

A core novelty of this study lies in leveraging the complementary information of four isotopes to enhance origin discrimination accuracy. Single-isotope models (AUC = 0.634–0.732) were limited by overlapping ranges, especially between marine samples, whereas the combined model (AUC = 0.901) leveraged the complementary strengths of each isotope. This aligns with recent food traceability literature emphasizing the value of multi-isotope approaches [44,45] an advances prior work by explicitly linking each isotope’s role to geographic environmental drivers—an advantage rooted in isotope ecology, where combining δ^13^C/δ^15^N (trophic/dietary tracers) with δ^2^H/δ^18^O (geographic/environmental tracers) provides orthogonal information that mitigates single-isotope limitations [18,46]. This is critical for *L*. *vannamei*, where dietary variability can mask weak geographic signals in δ^13^C/δ^15^N alone [2] and the framework remains robust in artificial aquaculture systems by capturing both aquaculture practices (e.g., feed type) and natural geographic characteristics (e.g., latitude, water type) [38], a combination that is the key to high-accuracy origin authentication for farmed aquatic products [12].

δ^13^C and δ^15^N further differentiated the tropical marine samples (Ecuador) from the domestic temperate/subtropical marine samples (Shandong, Fujian, Guangxi), a pattern linked to trophic dynamics [27]. The trophic dynamics-driven δ^15^N variability in marine shrimp is consistent with isotope ecology studies of coastal aquaculture systems, where dietary nitrogen assimilation is modulated by phytoplankton community structure and aquaculture feed composition [2,15], while the latitudinal trend of δ^2^H in shrimp tissues closely matched the global precipitation isotope pattern for East Asia and the tropical Pacific [16,17], confirming that aquatic organisms faithfully record the isotopic signature of ambient water sources from large-scale hydrological processes.

The LDA cross-validation accuracy of 81.3% is comparable to or exceeds that of previous studies on shrimp traceability, which often rely on more expensive techniques such as metabolomics or trace element analysis [22]. Furthermore, the binary logistic regression model achieved a high prediction rate for domestic samples (96.7%), which is valuable for regulatory purposes of verifying labeling authenticity. Partial overlap between the Domestic and Foreign groups may result from similarities in certain environmental factors: carbon and nitrogen isotopes are influenced by diet and trophic levels [15,38,40], and natural fluctuations in stable isotope ratios may also cause data overlap despite distinct sampling environments.

The explicit correlations between isotopes and geographic factors (Table 2) not only validate the model’s reliability but also offer ecological insights for shrimp aquaculture. Inner Mongolia’s distinct isotopic clustering (Figure 2, Figure 3, Figure 4 and Figure 5) underscores the model’s sensitivity to aquaculture water type; as an inland freshwater site, its shrimp had unique δ^13^C/δ^15^N signatures linked to terrestrial nutrient inputs [47], a finding with practical relevance: consumers often perceive freshwater shrimp as less flavorful than marine counterparts [48,49], and the four-isotope model can verify “freshwater vs. marine” labeling claims to support transparent marketing and consumer choice. For coastal aquaculture (Shandong, Fujian, Guangxi), δ^18^O’s strong association with evaporation intensity suggests potential applications in climate-adaptive farming. As global warming increases coastal evaporation [50,51], δ^18^O in shrimp may shift—monitoring δ^18^O in shrimp could track environmental changes and optimize water management. Similarly, δ^2^H’s latitudinal trend offers a “quick-screening tool” for origin verification: shrimp with δ^2^H > −90‰ are likely from low-latitude regions (e.g., Shandong), while values < −100‰ suggest high-latitude or inland origins (e.g., Inner Mongolia), reducing the need for full four-isotope analysis in preliminary screening.

Compared to multi-isotope studies that included sulfur (δ^34^S), our four-isotope model achieved comparable accuracy (~81% vs. Zhi et al.’s [5] ~85%) with fewer analytes. A recent multi-isotope traceability study for *L*. *vannamei* also highlighted that multi-isotope tracers are key to improving the accuracy and robustness of origin discrimination models [5]. This efficiency is notable: sulfur isotope analysis requires additional sample pretreatment and instrumentation [5], whereas δ^13^C/δ^15^N/δ^2^H/δ^18^O can be analyzed sequentially via EA-IRMS, reducing cost and analysis time and enabling faster, more cost-effective origin testing for industry-scale application.

### 4.3. Validation of Sample Pretreatment

Various drying protocols have been widely employed for stable isotope analysis, including freeze-drying, oven-drying at 50 °C for 25 h, 60 °C for 48 h, and 70 °C for 48 h [5,14,25,26,52]. In the present study, shrimp muscle samples were oven-dried at 55 °C for 48 h until constant weight (weight change < 0.001 g) to eliminate residual moisture. This moderate-temperature condition was selected to balance dehydration efficiency and isotopic integrity, especially for the more sensitive δ^2^H and δ^18^O signatures. Previous studies have demonstrated that mild to moderate drying/heating typically causes small or no shifts in δ^2^H/δ^18^O, but effects depend strongly on sample matrix, temperature intensity, and the binding state of water or organic hydrogen/oxygen [53]. For instance, in wheat noodles, low-temperature drying regimes (40–60 °C) maintained relatively stable δ^2^H and δ^18^O, while obvious isotopic shifts only occurred at higher temperatures (e.g., 80 °C) [53].

Although freeze-drying is often considered ideal for minimizing isotopic alteration, it is less accessible and time-consuming for routine traceability analysis [14]. Conversely, temperatures at or above 70 °C may increase the risk of unwanted isotopic fractionation or matrix degradation in certain biological materials. Repeated instrumental measurements further confirmed that dried samples exhibited excellent stability, with relative standard deviations (RSD) of less than 1% for both δ^2^H and δ^18^O values in this study. In addition, the permutation test results of the OPLS-DA model confirmed no overfitting, providing indirect evidence that the adopted drying conditions did not induce measurable isotopic shifts.

For lipid extraction, a chloroform/methanol (2:1, *v*/*v*) solution was used, which is a well-established and widely recommended defatting procedure in stable isotope analysis. This method has been extensively validated in the literature [14,26], confirming its scientific validity and compatibility with stable isotope analysis. Importantly, these reagents were used exclusively for sample defatting, and all shrimp specimens were naturally deceased after harvest and frozen before any laboratory pretreatment. Collectively, the optimized drying and defatting procedures ensure the accuracy, reliability, and comparability of isotopic data, fully supporting the robustness of the multi-stable isotope approach used for geographic origin authentication of *L*. *vannamei*.

### 4.4. Methodological Innovation

A key methodological contribution of this study is the integrated application of multi-stable isotope tracers (δ^13^C, δ^15^N, δ^2^H, δ^18^O) combined with a rigorous multivariate statistical analysis framework for shrimp geographic origin identification. This framework combines unsupervised exploratory analysis (PCA) with supervised discriminant models (LDA, OPLS-DA) and is supplemented by comprehensive statistical validation procedures to ensure the reliability and robustness of results. The selection of the four isotope indices takes full advantage of their distinct environmental response characteristics, enabling effective differentiation of shrimp from inland freshwater, domestic coastal, and foreign marine aquaculture areas—and filling the research gap of multi-origin discrimination for commercial shrimp using hydrogen and oxygen isotopes combined with carbon and nitrogen isotopes. Tailored for the isotopic traceability of *L*. *vannamei*, this framework accounts for the species-specific isotopic response to environmental and dietary factors [35,36], and aligns with the latest methodological requirements for high-accuracy shrimp origin authentication [5]. Beyond shrimp origin authentication, this methodological framework provides a standardized reference for the isotopic traceability of other aquatic products, with good scalability and application value in food traceability research.

### 4.5. Limitations and Future Directions

A key limitation of this study is the lack of consideration for seasonal variability in isotopic composition, which may restrict the model’s generalizability. This is a critical factor for *L*. *vannamei* traceability, as seasonal isotopic variations from dietary and environmental shifts, the strong coupling between shrimp tissue δ^2^H/δ^18^O and seasonally variable pond water isotopes, and the consequent reduction in model discrimination accuracy have all been empirically verified [5,35,36]. Previous research has shown that seasonal changes in environmental conditions and biological processes can significantly alter the stable isotope signatures of aquatic organisms [18,37,50]. For Pacific white shrimp, three main seasonal factors are likely to drive isotopic variability: (1) water temperature fluctuations that modify metabolic rates and isotopic fractionation of δ^13^C and δ^15^N during nutrient assimilation [37]; (2) seasonal shifts in feed availability—e.g., inland shrimp may rely more on artificial feeds in winter (enriching δ^13^C) and natural phytoplankton in summer (diluting δ^13^C), while coastal shrimp may be affected by marine phytoplankton blooms [18,50]; (3) precipitation/evaporation patterns that change the δ^2^H and δ^18^O signatures of aquaculture water, such as summer rainfall diluting δ^18^O or winter evaporation enhancing it [37]. These effects could lead to overlapping isotopic ranges between origins in non-summer seasons, reducing classification accuracy.

To address this, future research should conduct year-round sampling (spring–winter) across 2–3 years for all target origins. This will quantify seasonal isotopic variability and enable the establishment of “seasonal correction factors” to adjust model parameters, ensuring consistent accuracy across harvest seasons. Integrating seasonal data with environmental parameters (temperature, precipitation) will also help clarify the key drivers of isotopic shifts, strengthening the model’s applicability for year-round seafood origin authentication.

## 5. Conclusions

Origin authentication of Pacific white shrimp is critical in China to meet consumer demands for distinguishing domestic and foreign sources, as well as individual domestic origins. This study validated that a four-isotope (δ^13^C, δ^15^N, δ^2^H, δ^18^O) model combined with chemometric approaches reliably discriminates shrimp geographic origins, with δ^2^H and δ^18^O identified as novel key indicators for verification. Shrimp isotopic signatures are directly shaped by geographic environmental factors (latitude, climate, water type, regional geology), which are assimilated into organisms via feed and water uptake. This work establishes a copyright-compliant, cost-effective isotopic approach for shrimp origin traceability, which can improve the accuracy of product labeling and provide a scalable framework for isotopic traceability in other aquatic food products.

## Figures and Tables

**Table 3:** Linear discriminant formulas for the five shrimp groups based on C, N, H, and O stable isotopes.

Group	Formula
Shandong	−519.619 × *δ*^13^C + 137.699 × *δ*^15^N − 4.025 × *δ*^2^H + 10.062 × *δ*^18^O − 6683.406
Fujian	−511.724 × *δ*^13^C + 137.793 × *δ*^15^N − 5.103 × *δ*^2^H + 10.498 × *δ*^18^O − 6609.131
Guangxi	−504.793 × *δ*^13^C + 135.863 × *δ*^15^N − 5.471 × *δ*^2^H + 10.545 × *δ*^18^O − 6478.625
Ecuador	−516.991 × *δ*^13^C + 140.245 × *δ*^15^N − 4.698 × *δ*^2^H + 10.031 × *δ*^18^O − 6695.008
Inner Mongolia	−493.495 × *δ*^13^C + 135.338 × *δ*^15^N − 5.763 × *δ*^2^H + 9.553 × *δ*^18^O − 6232.473

**Table 4:** Cross-validation AUC and optimal thresholds for individual factors and the combined Model.

Factor	AUC	95% CI (AUC)	Best_Thresholds (Specificities, Sensitivities)
C (‰)	0.732	0.655–0.809	−22.8735 (0.517, 1)
N (‰)	0.671	0.569–0.773	5.311 (0.617, 0.8)
H (‰)	0.708	0.627–0.788	−99.35 (0.517, 1)
O (‰)	0.634	0.549–0.719	20.425 (0.433, 0.967)
Combined Model	0.901	0.819–0.983	0.36483 (0.942, 0.833)

Note: 95% CI = 95% confidence interval. No zero values were observed in the 95% CI of all parameters, indicating statistical significance (*p* < 0.05).

**Table 5:** Linear discriminant classification results for the five shrimp groups based on C, N, H, and O stable isotopes. Accuracy (%) indicates average accuracy across locations.

Discriminant Analysis Type	Group	Shandong	Fujian	Guangxi	Ecuador	Inner Mongolia	Accuracy (%)
Original Classification (%)	Shandong	76.7	0	0	23.3	0	83.3
	Fujian	0	80	10	10	0	
	Guangxi	0	16.7	76.7	0	6.7	
	Ecuador	13.3	3.3	0	83.3	0	
	Inner Mongolia	0	0	0	0	100.0	
Cross-validation Classification (%)	Shandong	76.7	0	0	23.3	0	81.3
	Fujian	0	76	10	13.3	0	
	Guangxi	0	23.3	70	0	6.7	
	Ecuador	13.3	3.3	0	83.3	0	
	Inner Mongolia	0	0	0	0	100	

**Table 6:** Cross-validation confusion matrix and overall classification accuracy.

Observed Value		Predicted Value		Accuracy
		VAR00005		
		0	1	
VAR00005	0	116	4	96.7
	1	12	18	60
Population Percentage				89.3

Note: VAR00005 is a categorical assignment variable for the geographical origin of shrimp samples, where VAR00005 = 0 represents domestic shrimp samples (Shandong, Fujian, Guangxi, Inner Mongolia) and VAR00005 = 1 represents foreign shrimp samples (Ecuador).

## Data Availability

The original contributions presented in this study are included in the article/Supplementary Material. Further inquiries can be directed to the corresponding author.

## Supplementary Materials

The following supporting information can be downloaded at: https://www.mdpi.com/article/10.3390/foods15081274/s1, Figure S1 Validation of OPLS-DA models for shrimp meat from different sorigins based on δ^13^C, δ^15^N, δ^2^H, and δ^18^O values: (A) Shandong; (B) Fujian; (C) Guangxi; (D) Ecuador; (E) Inner Mongolia.
