# Supplementary material for: C, H, O, N Stable Isotope Analysis Coupled with Chemometrics for Geographic Origin Authentication of Pacific White Shrimp (Litopenaeus vannamei) in China
Source: Foods. 2026 Apr 8;15(8):1274. doi: 10.3390/foods15081274 (PMC13115197; doi:10.3390/foods15081274)
Supplement: Supplementary file 1 [file foods-15-01274-s001.zip › foods-4149838-supplementary.pdf]

Figure S1 shows the permutation test validation of OPLS-DA models for shrimp meat from different origins based on $\delta^{13}C$, $\delta^{15}N$, $\delta^{2}H$, and $\delta^{18}O$ values.

(A)

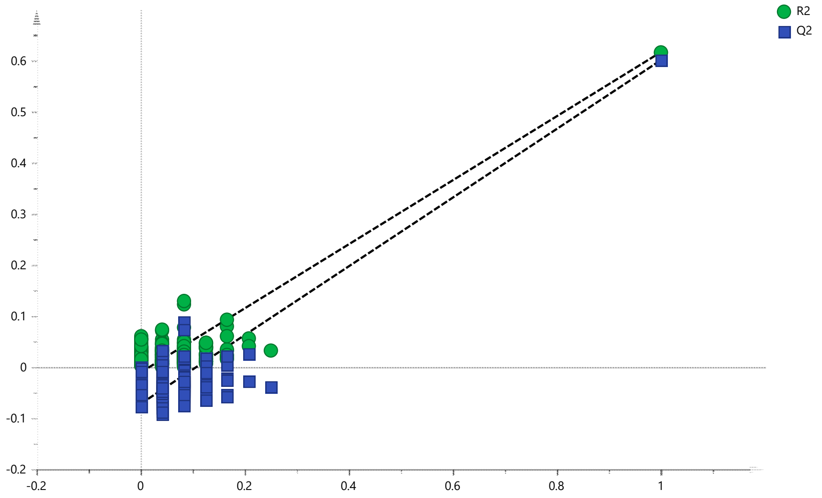


(B)

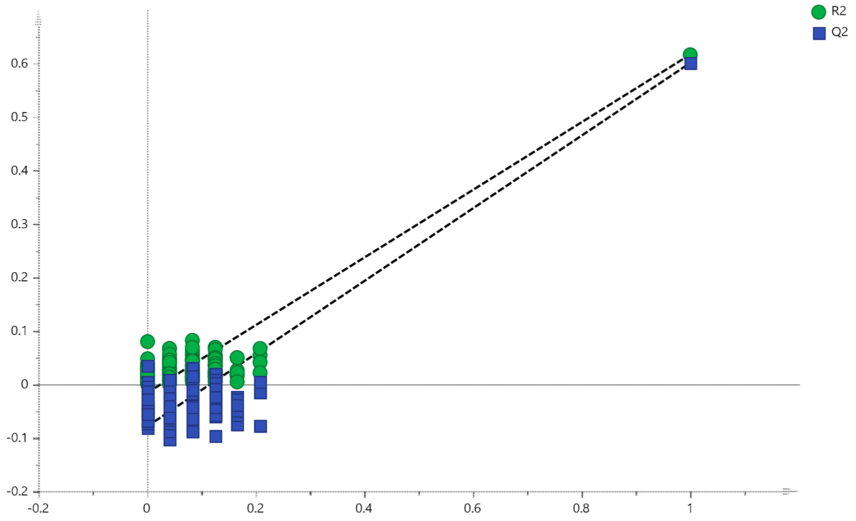


(C)

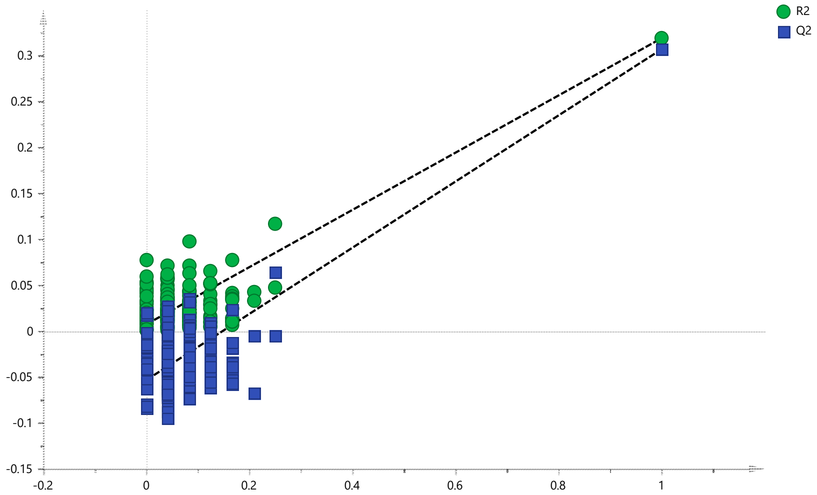


(D)

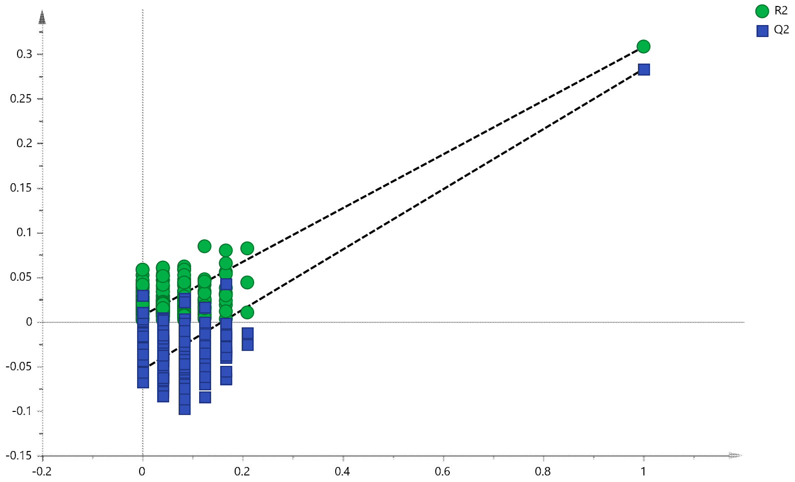


(E)

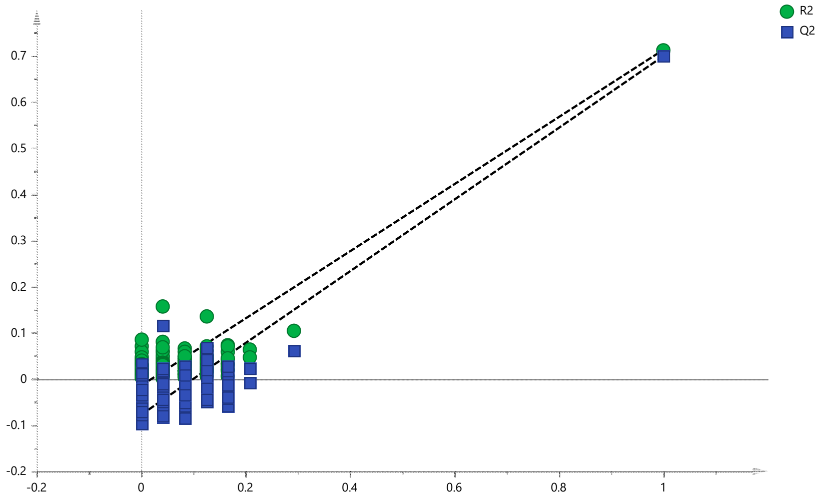


Figure S1 Validation of OPLS-DA models for shrimp meat from different origins

based on $\delta^{13}C$, $\delta^{15}N$, $\delta^{2}H$, and $\delta^{18}O$ values: (A) Shandong; (B) Fujian; (C) Guangxi; (D) Ecuador; (E) Inner Mongolia.
